# Selective detection of trinitrophenol by a Cd(ii)-based coordination compound†

**DOI:** 10.1039/c9ra08614e

**Published:** 2019-11-26

**Authors:** Basudeb Dutta, Rakesh Purkait, Suprava Bhunia, Samim Khan, Chittaranjan Sinha, Mohammad Hedayetullah Mir

**Affiliations:** Department of Chemistry, Aliah University New Town Kolkata 700 156 India chmmir@gmail.com; Department of Chemistry, Jadavpur University Jadavpur Kolkata 700 032 India

## Abstract

A Cd(ii)-based coordination compound, [CdI_2_(4-nvp)_2_] (1), has been synthesized using CdI_2_ and monodentate N-donor ligand 4-(1-naphthylvinyl)pyridine (4-nvp). The solid-state supramolecular architecture has been characterized by X-ray crystallography. An acute thermal stability and excellent level of phase purity tempted us to use it for material applications. Interestingly, compound 1 exhibits a high selectivity towards trinitrophenol (TNP) in the presence of other nitroaromatics. Therefore, this material may be used for anti-terrorist activities in the detection of explosive materials as well as in the recognition of TNP in analytical laboratories.

## Abstract 2

A Cd(ii)-based coordination compound, [CdI_2_(4-nvp)_2_] (1), has been synthesized using CdI_2_ and monodentate N-donor ligand 4-(1-naphthylvinyl)pyridine (4-nvp).

## Graphical abstract

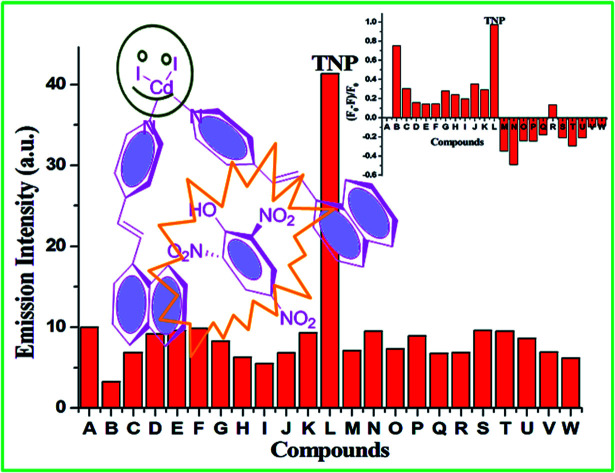

## Introduction

Synthetic inorganic chemistry is being enriched every day through the generation of various types of coordination compounds.^1–3^ In the past, it was a really difficult task to authenticate and/or characterize coordination moieties. However, after the historic invention of Alfred Werner in the year 1913, the chemistry of these compounds attracted the attention of the research community. Inorganic metal ions and organic ligands are combined to form coordination compounds due to their inherent electronic properties. The desired structural architectures have been achieved *via* judicious selection of metal ions and ligands. The chemistry of such materials is of great interest to synthetic chemists because of their intriguing structural motifs as well as potential applications in areas such as catalysis, magnetism, ion exchange, drug delivery, conductance, photoluminescence and chemical sensing.^4–15^ In addition to the direct role of the metal ions and organic ligands, there are many more essential conditions, including counter anions, reaction temperatures, solvent media, external stimuli, and pH of the reaction medium, which play crucial roles during compound formation. In addition, there is an eternal relationship among the structure, property and application of the compounds. Therefore, one can easily design the compound according to the desired application.^16–19^

In recent years, the design of chemical sensors and their applications in the detection of ions/molecules have received active interest from chemistry, chemical engineering, physics, electrical and electronic engineering, and many other branches of science and technology. Usually, a molecular sensor is a chemical compound (organic or inorganic complex) that is used for sensing an analyte to crop a detectable change or signal. The action of a chemosensor typically involves the continuous monitoring of the activity of a chemical species in a given matrix such as solution, air, blood, tissue, waste effluents, and drinking water.^20,21^ The application of a chemosensor is designated as chemosensing, which is generally a form of molecular recognition. All chemosensors are intended to comprise a signalling moiety and a recognition moiety and they are connected either directly or through a connector or a spacer.^21,22^

Nitroaromatics^22–29^ are normally explosive in nature and used in terroristic activities. They have become an area of concern for the Crime Bureau of Intelligence (CBI), Ministry of Home Affairs (MHA), and Ministry of Defense (MOD) of the government.^16,17^ Many methods of detecting explosive materials such as energy-dispersive X-ray diffraction, police dog detection, ion migration spectroscopy, plasma desorption mass spectrometry, surface-enhanced Raman spectroscopy, and additional imaging techniques are known. However, none of them are economically viable. Therefore, it is very important to develop a special type of chemosensor that is efficient, commercially realistic, rapidly responsive, inexpensive and portable. In addition, there is a possibility of mixing aromatic compounds in the laboratory or in industry. Therefore, it is necessary to easily detect them at a glance and quantify. Keeping the above mentioned points in mind, we have designed and synthesized a Cd(ii)-based discrete coordination compound [CdI_2_(4-nvp)_2_] (1), (4-nvp = 4-(1-naphthylvinyl)pyridine), which is highly selective towards trinitrophenol (TNP).

## Experimental section

### Materials and physical method

All chemicals were obtained in reagent grade and were used without any additional purification. For the analysis of elements, *i.e.* carbon, hydrogen and nitrogen, a PerkinElmer 240C elemental analyzer was used. Thermal gravimetric analysis (TGA) was performed using a PerkinElmer Pyris Diamond TG/DTA instrument in a temperature range between 30 °C and 800 °C under an inert nitrogen atmosphere at a heating rate of 10 °C min^−1^. The powder XRD data of the finely powdered sample was collected on a Bruker D8 Advance X-ray diffractometer using Cu Kα radiation (*λ* = 1.548 Å) produced at 40 kV and 40 mA. To verify the phase purity of the sample, the PXRD spectrum was recorded in a 2*θ* range of 5–50°. The fluorescence spectra were recorded using a PerkinElmer spectrofluorimeter model LS55. The UV-vis spectra were obtained from a PerkinElmer Lambda 25 spectrophotometer. The time-resolved single-photon counting measurements were performed using a time-correlated single-photon counting setup from HORIBA Jobin-Yvon. ^1^H NMR spectra were collected in DMSO-d_6_ using a Bruker 300 MHz FT-NMR spectrometer with TMS as the internal standard.

### Synthesis of compound 1

A solution of 4-nvp (0.046 g, 0.2 mmol) in MeOH (2 mL) was slowly and carefully layered into a solution of CdI_2_ (0.073 g, 0.2 mmol) in H_2_O (2 mL) using 2 mL of a 1 : 1 (v/v) buffer solution of MeOH and H_2_O. It was then allowed to diffuse for a few days. The colorless needle-shaped crystals of [CdI_2_(4-nvp)_2_] (1) were obtained after three days (0.107 g, yield 65%). Elemental analysis (%) calcd for C_34_H_26_CdI_2_N_2_: C 49.27, H 3.16, N 3.18; found: C 49.31, H 3.13, N 3.41.

### General X-ray crystallography

A suitable single crystal of compound 1 with the proper dimensions (0.124 × 0.105 × 0.099 mm^3^) was used for data collection using a Bruker SMART APEX II diffractometer equipped with graphite-monochromated MoKα radiation (*λ* = 0.71073 Å). The molecular structure of the single crystal was solved using the SHELX-97 package.^30^ Non-hydrogen atoms of the compound were refined with anisotropic thermal parameters. All the hydrogen atoms were located in their geometrically perfect positions and constrained to ride on their parent atoms. The crystallographic data for compound 1 is summarized in Table S1.† The selected bond lengths and bond angles are also given in Table S2.†

### Hirshfeld surfaces analysis

Hirshfeld surfaces^31–33^ and the associated two-dimensional (2D) fingerprint^34–36^ plots were calculated using Crystal Explorer,^37^ with the bond lengths to the hydrogen atoms being set to standard values.^38^ For each point on the Hirshfeld isosurface, two distances, *d*_e_ (the distance from the point to the nearest nucleus external to the surface) and *d*_i_ (the distance to the nearest nucleus internal to the surface), were defined. The normalized contact distance (*d*_norm_) based on *d*_e_ and *d*_i_ is given by
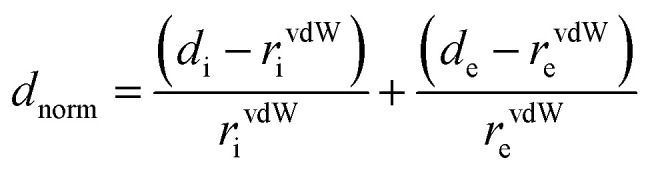
where *r*^vdW^_i_ and *r*^vdW^_e_ are the van der Waals radii of the atoms. The value of *d*_norm_ is negative or positive depending on the intermolecular contacts being shorter or longer than the van der Waals separations. The parameter *d*_norm_ displays a surface with a red-white-blue color scheme, where the bright red spots highlight shorter contacts, the white areas represent contacts around the van der Waals separation, and the blue regions are devoid of close contacts. For a given crystal structure and set of spherical atomic electron densities, the Hirshfeld surface is unique,^39^ and thus suggests the possibility of gaining additional insight into the intermolecular interaction of molecular crystals.

### Theoretical calculations

By utilizing the GAUSSIAN-09 ^40^ program package, the optimized geometries and molecular functions of the compound were attained. The hybrid DFT-B3LYP^41^ theoretical functional was used throughout the process. The LanL2DZ basis set was allotted for the compound. The single crystal X-ray coordinates were taken for 1. To entrust the low lying electronic transitions in the spectra, the time-dependent density functional theory (TDDFT)^42–44^ formalism of the compound was developed. To calculate the fractional involvement of the metal molecular orbitals and organic ligand molecular orbitals, Gauss sum^45^ was operated.

## Results and discussion

### Structural descriptions of [CdI_2_(4-nvp)_2_] (1)

**Fig. 1:**
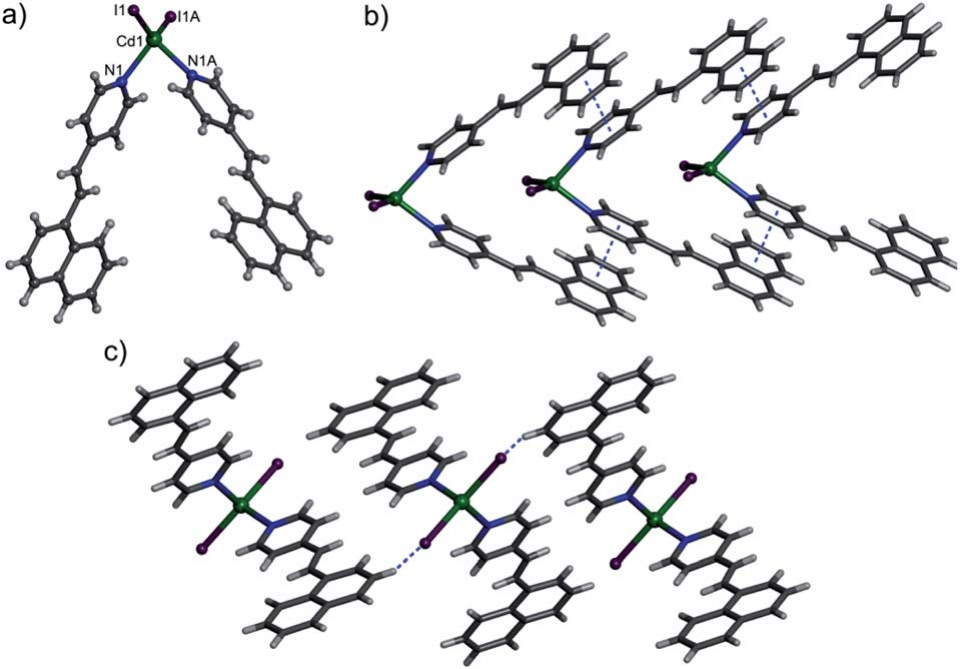
(a) A representation of 1 showing the coordination environment of the Cd(ii) centre. (b) The 1D chain formed by π⋯π stacking interactions. (c) C–H⋯I interactions in 1.

X-ray crystallography analysis revealed that 1 crystallizes in the *P*1̄ space group with *Z* = 4. The asymmetric unit contains a 4-nvp ligand, a Cd(ii) ion, and an iodide ion. The coordination geometry around the Cd(ii) ion is tetrahedral and bonded to two 4-nvp ligands through pyridine N atoms and two iodide ions (Fig. 1a). In 1, the Cd–N and Cd–I bond distances are 2.306(4) and 2.6915(4), respectively. However, the 4-nvp ligands are not exactly planar. The dihedral angle between the planes of pyridine and the aryl rings is 5.54°. In the crystal structure, the discrete neutral [CdI_2_(4-nvp)_2_] units stacked together by the combination of π⋯π stacking (Fig. 1b) and C–H⋯I (Fig. 1c) interactions to generate a supramolecular assembly.

### Hirshfeld surface analysis of 1

**Fig. 2:**
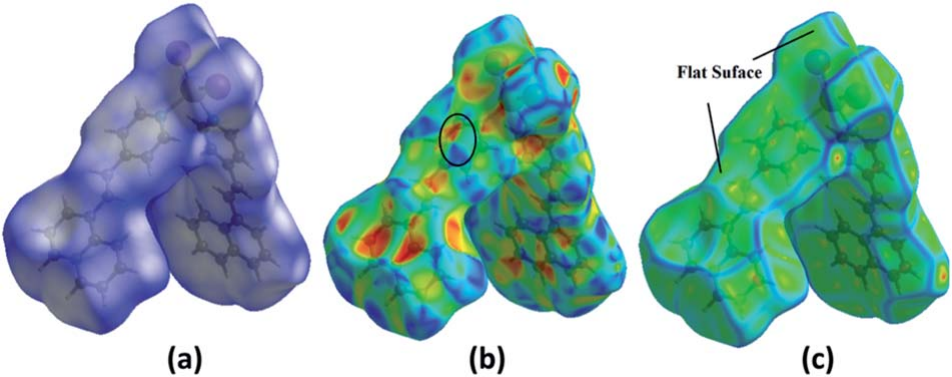
Hirshfeld surfaces mapped with (a) *d*_norm_ and (b) shape index (the presence of red and blue triangles is shown in the black ellipse, in which the red and blue color represent the bumps and hollow regions on the shape index surfaces, respectively). (c) Curvedness of compound 1 for identifying the planar (green) and curved (blue edge) regions for planar stacking interactions.

**Fig. 3:**
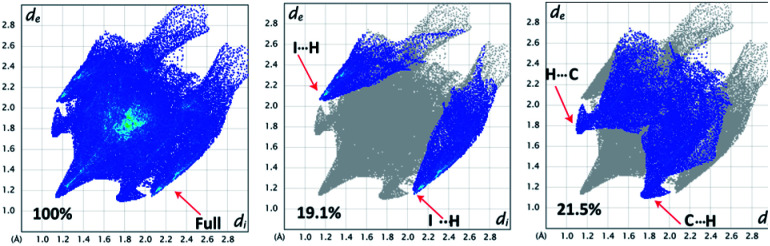
2D fingerprint plots: full (left), I⋯H/H⋯I (middle), and C⋯H/H⋯C (right) interactions that contributed to the total Hirshfeld surface area for compound 1.

The Hirshfeld surfaces for 1 are mapped over the *d*_norm_, shape index and curvedness (Fig. 2). The surfaces are shown as transparent to allow the visualization of the molecular moiety, around which they were calculated. The dominant interactions are between the C and H atoms for 1. Other visible spots in the Hirshfeld surfaces correspond to the H⋯H contacts. The small extent of the area and light color on the surface indicates a weaker and longer contact other than hydrogen bonds. The C⋯H/H⋯C interactions appear as distinct spikes in the 2D fingerprint plot (Fig. 3). The complementary regions are visible in the fingerprint plots, where one molecule acts as a donor (*d*_e_ > *d*_i_) and the other as an acceptor (*d*_e_ < *d*_i_). The fingerprint plots can be decomposed to highlight the contributions from different interaction types, which overlap in the full fingerprint.^34^

The proportion of C⋯H/H⋯C interactions comprise 21.5% of the interactions in 1. The C⋯H interaction is represented by a lower spike (*d*_i_ = 1.74, *d*_e_ = 1.16 Å) and the H⋯C interaction is also represented by a lower spike (*d*_i_ = 1.16, *d*_e_ = 1.74 Å) (Fig. 3) and can be viewed as bright red spots on the *d*_norm_ surface (Fig. 2). The proportions of the I⋯H/H⋯I interactions comprise 19.1% of the Hirshfeld surfaces for each molecule of the complex. The I⋯H interaction is represented by a lower spike (*d*_i_ = 2.06, *d*_e_ = 1.14 Å) and the H⋯I interaction is represented by another upper spike (*d*_i_ = 1.14, *d*_e_ = 2.06 Å) spike (Fig. 3) and can be viewed as bright red spots on the *d*_norm_ surface (Fig. 2). The shape index represents the local morphology of any given surface in terms of colour coded information, *i.e.*, hollow (red) and bumps (blue). Fig. 2b and c show how the shape index and curvedness surfaces are used to identify planar π⋯π stacking interactions. The presence of red and blue triangles in the same region of the shape index surface shown by the black ellipse in Fig. 2b indicates that the π⋯π interaction is almost identically present in the crystal structure. Blue triangles represent the convex region, which is formed due to the carbon atoms present in the naphthalene ring of the molecule inside the surface, while red triangles represent concave regions due to the carbon atoms of the π-stacked molecule above it. The mapping of the curvedness on the Hirshfeld surface (Fig. 2c) shows a flat green region separated by blue edges. These clearly visible flat regions on the curvedness surface are another characteristic of the π⋯π stacking interaction. Compound 1 has delocalized π-electrons in the C=C bonds, due to which all C=C bonds can have different energy spacings between the ground and excited states, which are responsible for radiative recombination, leading to a luminescence spectrum for the grown crystal. Therefore, compound 1 can be a good candidate for sensing applications.

### Sensor application

**Fig. 4:**
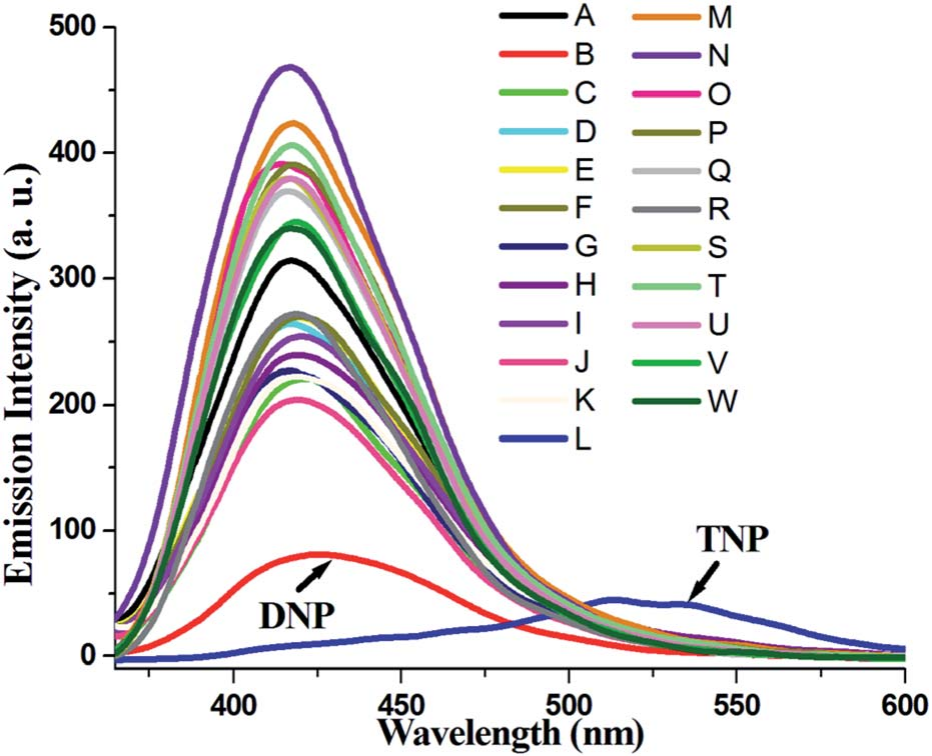
Fluorescence spectra of 1 in the presence of various nitroaromatics (A: 1, B: DNP, C: nitrophenol, D: nitrobenzoic acid, E: dinitrobenzene, F: nitrotoluene, G: dinitrophenol, H: nitrosalisylic acid, I: chloronitro benzene, J: 4-nitrophenol, K: nitrocoumarine, L: TNP, M: *p*-cresol, N: 2,4-dichloro phenol, O: 4-chloro-3-methyl phenol, P: 2-iodo benzoic acid, Q: 4-chlorophenol, R: *o*-vaniline, S: 4-chloroaniline, T: 4-methoxyphenol, U: *p*-xylene, V: diphenylamine, and W: 2,6-ditertyarybutyl*p*-cresol) in acetonitrile. (*λ*_ex_: 320 nm; excitation slit: 15; emission slit: 10).

**Fig. 5:**
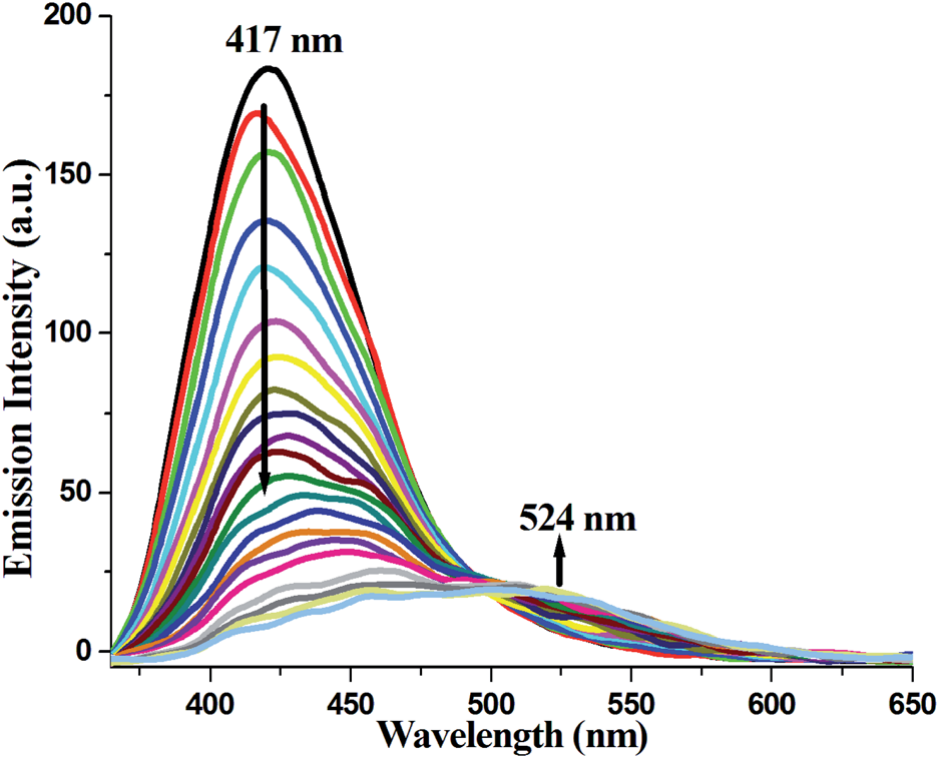
Changes in the fluorescence emission spectra of the Cd-complex on the gradual addition of TNP in acetonitrile medium.

**Fig. 6:**
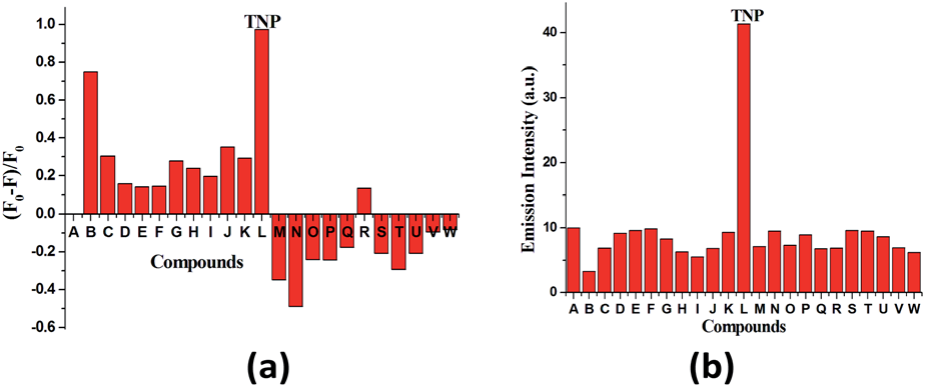
(a) Fluorescence quenching efficiency of the mentioned aromatic compound monitored at 417 nm (A to W as Fig. 4). (b) Fluorescence emission intensities at 524 nm of 1 in the presence of the mentioned aromatics (A to W as Fig. 4).

**Fig. 7:**
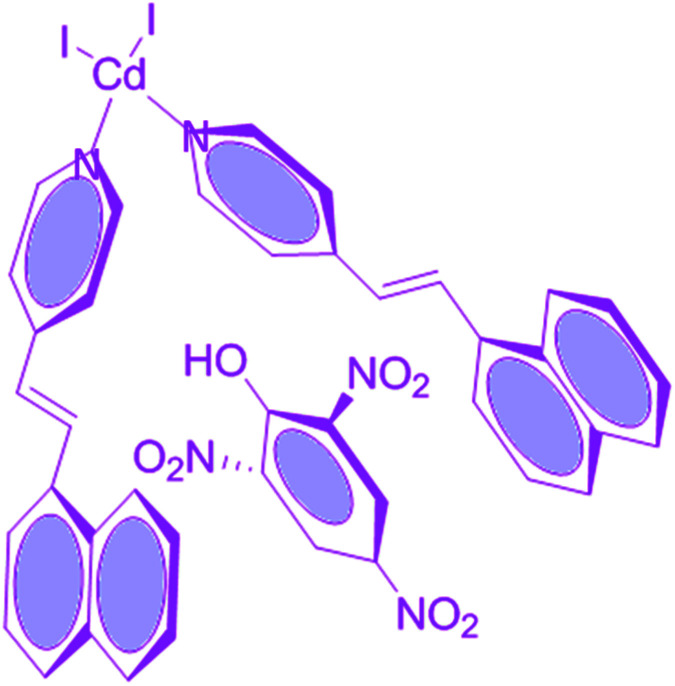
Possibilities for the π-interaction of TNP with the Cd-complex.

**Fig. 8:**
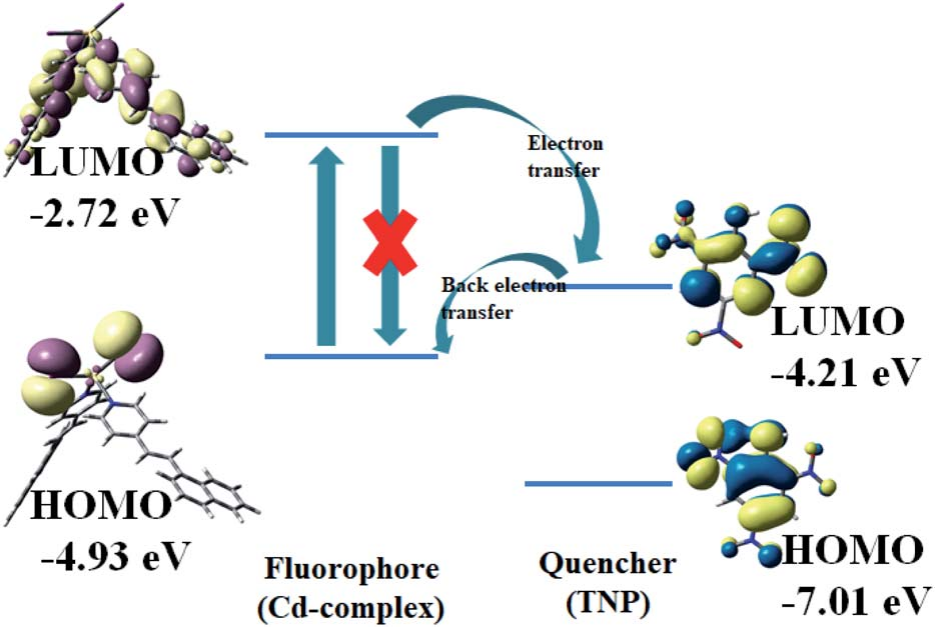
Frontier orbital energy correlation diagram *via* electron transfer fluorescence quenching.

Compound 1 in acetonitrile shows two absorption bands at 222 and 318 nm (Fig. S3†). On fixing the excitation wavelength at 320 nm, the compound exhibits strong fluorescence at 417 nm (Fig. S4†). Fluorescence spectra of compound 1 in acetonitrile after mixing with 23 different aromatic compounds exhibit that the highest quenching is observed in the presence of TNP (Fig. 4). It is very astonishing to see a new emission band centered at *λ*_em_ = 524, developed in the presence of a higher concentration of TNP exclusively, which shifted in the red region by 107 nm compared to that for the free compound 1 (*λ*_em_, 417 nm) (Fig. 5). Thus, compound 1 is very selective towards TNP, as confirmed by the fluorescence spectroscopy results in terms of both quenching at 417 nm (Fig. 6a) as well as the turn-on at 524 nm (Fig. 6b). Nitroaromatics are oxidizers because of the presence of low-lying unoccupied π*-orbitals, which can accept an electron from the excited state fluorophore, thus efficiently turning off the fluorescence emission of this compound.^35^ For the fruitful fluorescence quenching of small toxic compounds such as nitroaromatic explosive compounds (NACs), it is necessary for them to be closer to the sensor molecule and interact with the sensor. The interactions are mainly based on π-interactions, namely, C–H⋯π and π⋯π stacking interactions.^36^ Herein, the presence of polyaromatic rings in emissive compound 1 makes it electron rich and the structure shows the possibility to form a good π⋯π stacked charged transfer complex (Fig. 7). The DFT computation using the B3LYP/LanL2DZ method for compound 1 resulted in LUMO (LUMO_Cd_) and HOMO (HOMO_Cd_) energy of −2.72 and −4.93 eV, respectively. However, the LUMO of TNP (LUMO_TNP_) was −4.21 eV, which was lower than LUMO_Cd_, but higher than HOMO_Cd_. On excitation, the electrons went from the HOMO_Cd_ to the LUMO_Cd_ and were unable to revert back due to the presence of the low-lying LUMO_TNP_. Thus, the electron jumps from the LUMO_Cd_ to the LUMO_TNP_ and then comes to the ground state (Fig. 8). Therefore, the quenching of the emission (*λ*_em_, 417 nm) for 1 has been observed for TNP and the structure of the compound encourages TNP to come closer to 1 through π⋯π interactions (Fig. 7). The fluorescence decay profiles of both 1 and 1 with TNP exhibit bi-exponential nature. The fluorescence lifetime of 1 is 1.06 ns, which decreased to 0.11 ns in the presence of TNP (Fig. S5†). A red-shifted turn-on band observed at a higher concentration of TNP may have been observed due to the formation of a co-complex between TNP and 1, which resulted in a new band gap corresponding to the emission at 524 nm.

In order to conclude the sensing and interaction mechanism of the nitroaromatics with the complex in solution, ^1^H NMR spectra have been recorded for varying amounts of TNP in DMSO-d_6_ (Fig. S6 and S7†). It was detected that the protons of the aromatic region gradually shifted to the downfield area with an increase in the concentration of the nitroaromatic compound. As we know, nitroaromatics are electron deficient and hence, during the formation of the pi-complex with these components (compound and nitroaromatics), the nitrocompounds withdraw the electron density from the complex and shift the protons toward the deshielded region. The other peaks remain practically at the same position in each step of this NMR titration. It was also confirmed that compound 1 was not decomposed during the interaction with the nitroaromatics. To understand the mechanism, fluorescence lifetime measurements for the sensor in the presence and absence of the quencher were performed. The fluorescence intensity ratio (*I*_0_/*I*) was plotted against the concentration of TNP and a Stern–Volmer (SV) plot was obtained (Fig. S8†). The SV coefficient, *K*_sv_, value was determined as 1.8 × 10^5^ M^−1^, which was due to the quenching of the fluorescence intensity. To quantify the sensing efficiency, the limit of detection (LOD) was calculated as 16.55 × 10^−7^ M from the 3*σ* method (Fig. S9†).^46^

## Conclusions

In conclusion, the 4-NVP coordinated Cd(ii)-based coordination compound was synthesized, and the molecular arrangement was assigned from a single crystal X-ray study. Phase purity and excellent thermal stability were also realized *via* corresponding PXRD and TGA study. The exceptional emission of compound 1 tempted us to perform the sensing experiment for the explosive TNP. Interestingly, in the presence of different aromatic compounds, 1 can easily detect TNP. Thus, compound 1 can be an outstanding material for detecting TNP during security checking.

## Conflicts of interest

There are no conflicts to declare.

## Supplementary Material

RA-009-C9RA08614E-s001

RA-009-C9RA08614E-s002
