# Supplementary material for: Selective detection of trinitrophenol by a Cd(ii)-based coordination compound
Source: RSC Adv. 2019 Nov 26;9(66):38718–23. doi: 10.1039/c9ra08614e (PMC9075976; doi:10.1039/c9ra08614e)
Supplement: RA-009-C9RA08614E-s001 [file RA-009-C9RA08614E-s001.pdf]

Electronic Supplementary Material (ESI) for RSC Advances.
This journal is © The Royal Society of Chemistry 2019

Selective detection of trinitrophenol by a Cd(II)-based coordination compound

Basudeb Dutta,[a] Rakesh Purkait,[b] Suprava Bhunia,[b] Samim Khan,[a] Chittaranjan Sinha,[b] and Mohammad Hedayetullah Mir*[a]

[a]*Department of Chemistry, Aliah University, New Town, Kolkata 700 156, India. Email: chmmir@gmail.com*

[b]*Department of Chemistry, Jadavpur University, Jadavpur, Kolkata 700 032, India.*

Supporting Information

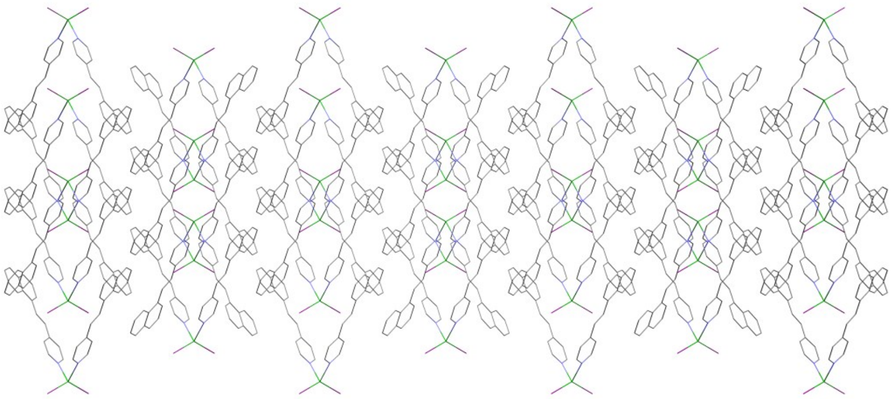

Fig. S1 3D supramolecular assembly of compound **1** viewed along *c*-axis.

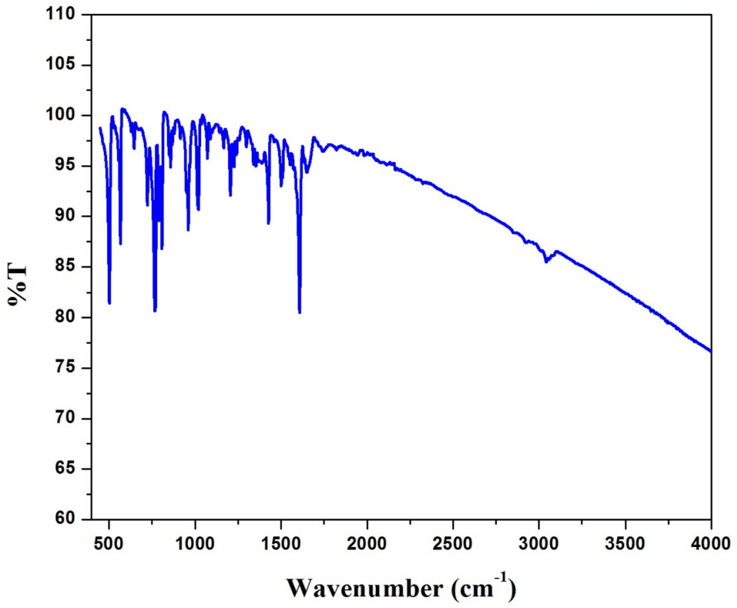

Fig. S2 FT-IR spectrum of compound **1**.

Table S1. Crystal data and refinement parameters for compound **1**

Formula	$C_{34}H_{26}CdI_2N_2$ (**1**)
fw	828.78
crystsyst	monoclinic
space group	*C*2/*c*
a (Å)	28.4498(19)
b (Å)	7.4390(5)
c (Å)	17.7851(12)
α(deg)	90
β(deg)	125.191(2)
γ (deg)	90
V (Å^3)	3076.1(4)
Z	4
D_{calcd}(g/cm^3)	1.790
μ(mm^{-1})	2.742
λ(Å)	0.71073
data[$I > 2\sigma(I)$]/params	2703/177
GOF on F^2	1.088
final *R* indices[$I > 2\sigma(I)$]^{a,b}	$R1 = 0.0329$<br>$wR2 = 0.0887$

$^aR1 = \Sigma||F_o| \quad |F_c||/ \Sigma|F_o|$, $^bwR2 = [\Sigma w(F_o^2 \quad F_c^2)^2/\Sigma w(F_o^2)^2]^{1/2}$

Table S2. Selected bond lengths and bond angles in **1**

I(1) - Cd(1)	2.6915(4)	Cd(1)-N(1)-C(5)-C(4)	166.6(5)
Cd(1) - N(1)	2.306(4)	C(3)-C(4)-C(5)-N(1)	1.0(10)
Cd(1) - N(1)a	2.306(4)	Cd(1)-N(1)-C(1)-H(1)	12
I(1) - Cd(1) - N(1)	105.24(11)	C(1)-N(1)-C(5)-H(5)	179
N(1) - Cd(1) - I(1)a	110.10(11)	N(1)a-Cd(1)-N(1)-C(1)	63.7(4)
Cd(1) - N(1) - C(1)	118.4(3)	I(1)-Cd(1)-N(1)-C(5)	5.5(5)
I(1) - Cd(1) - I(1)a	131.34(2)	I(1)a-Cd(1)-N(1)-C(5)	151.4(4)
N(1)- Cd(1) - N(1)a	85.22(16)	I(1)-Cd(1)-N(1)-C(1)	173.3(4)
Cd(1) - N(1) - C(5)	123.3(4)	I(1)a - Cd(1) - N(1)a	105.24(11)
I(1) - Cd(1) - N(1)a	110.10(11)	C(15)-C(16)-C(17)-H(17)	178

Symmetry Code: a = 1-x, y, 1/2-z

Table 3. Energy of different MOs from DFT calculation

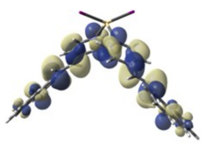	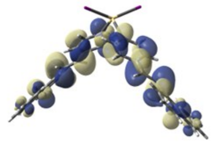	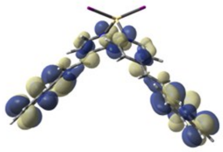
LUMO<br>-2.72eV	LUMO+1<br>-2.7eV	LUMO+2<br>-1.7eV
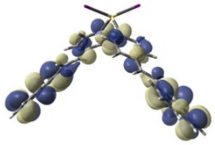	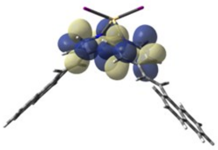	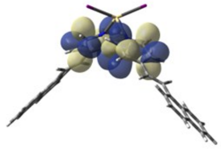
LUMO+3<br>-1.66eV	LUMO+4<br>-1.6eV	LUMO+5<br>-1.52eV
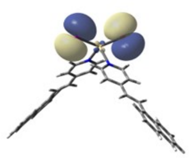	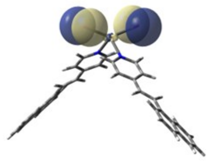	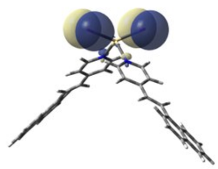
HOMO<br>-4.93eV	HOMO-1<br>-5.23eV	HOMO-2<br>-5.44eV
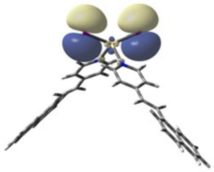	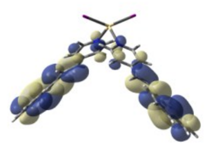	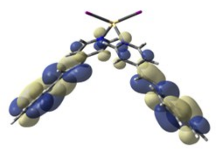
HOMO-3<br>-5.51eV	HOMO-4<br>-5.86eV	HOMO-5<br>-6.17eV

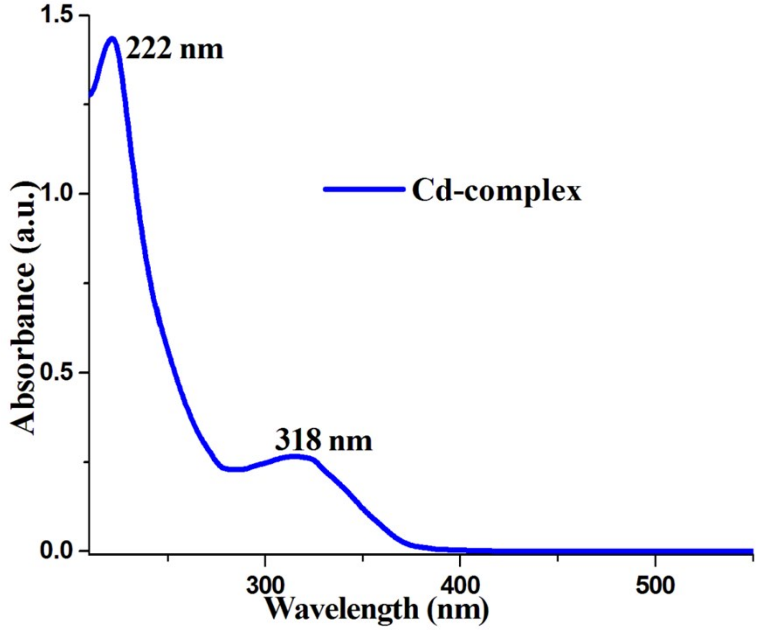

Fig. S3 UV-vis spectrum of **1** in acetonitrile.

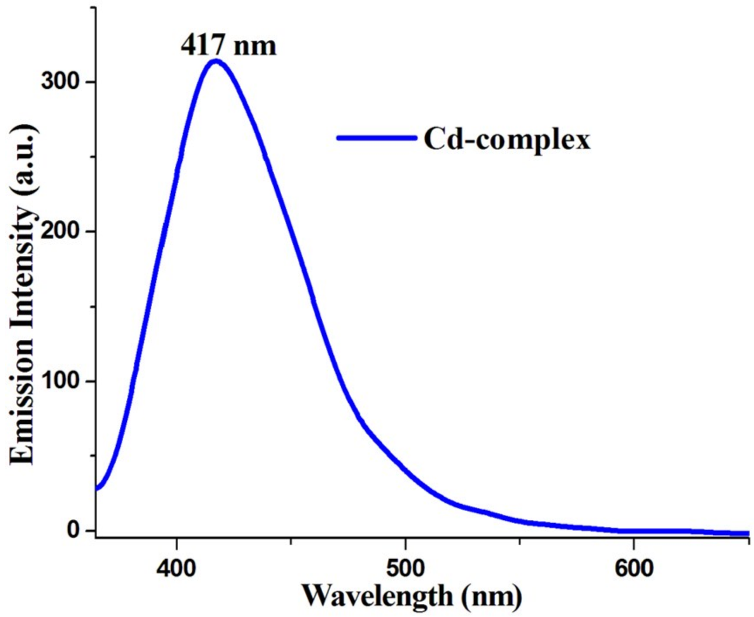

Fig. S4 Fluorescence spectrum of **1** in acetonitrile (λ_{ex} 320).

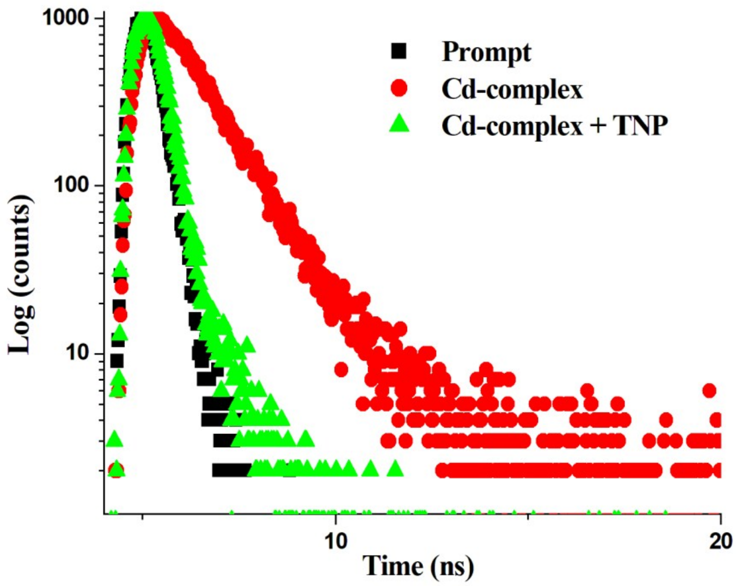

Fig. S5 Fluorescence decay profile of compound **1** and compound **1** with TNP.

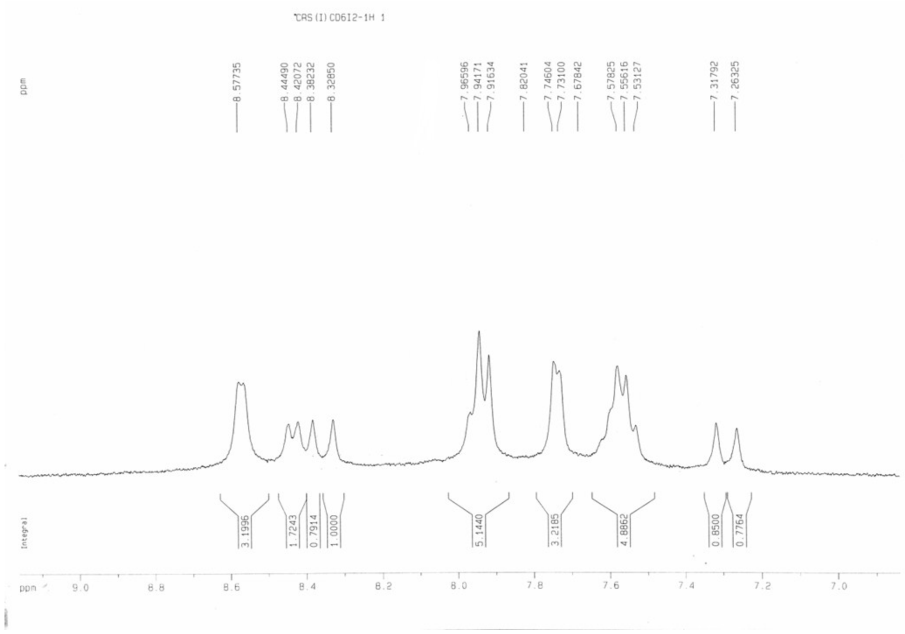

Fig. S6 ^{1}H NMR spectrum of **1** in DMSO-d_6 solvent.

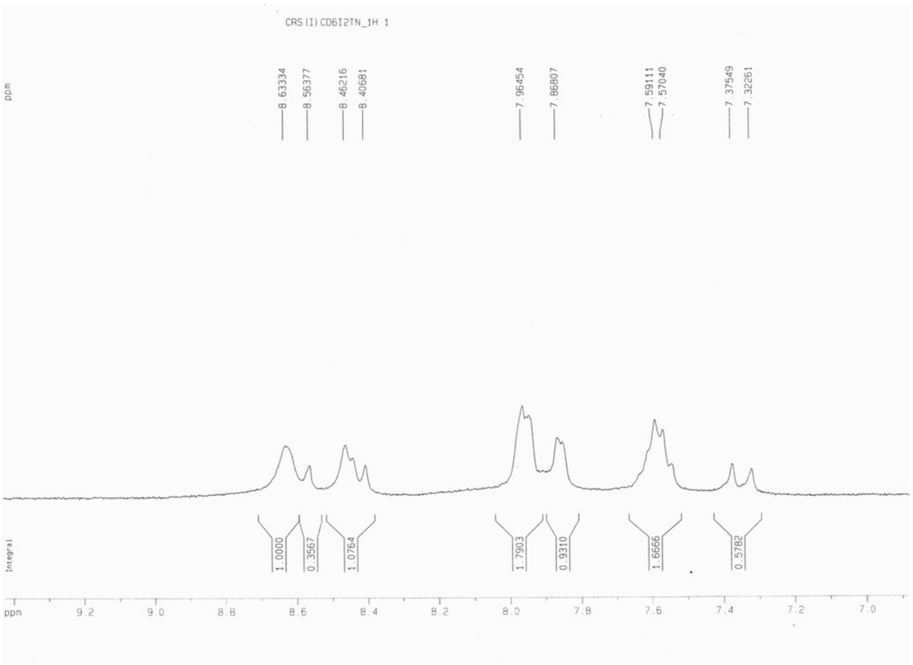

Fig. S7 ^{1}H NMR spectrum of **1** with trinitrophenol (TNP) in DMSO-d_6.

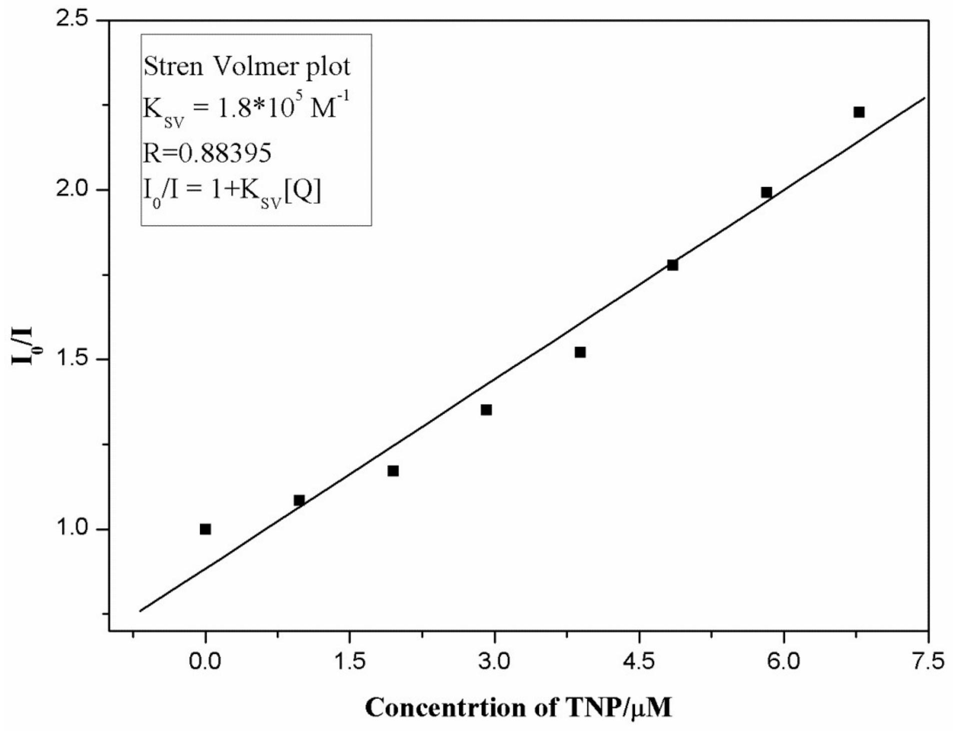

Fig. S8 Plot of I_0/I against the concentration of TNP.

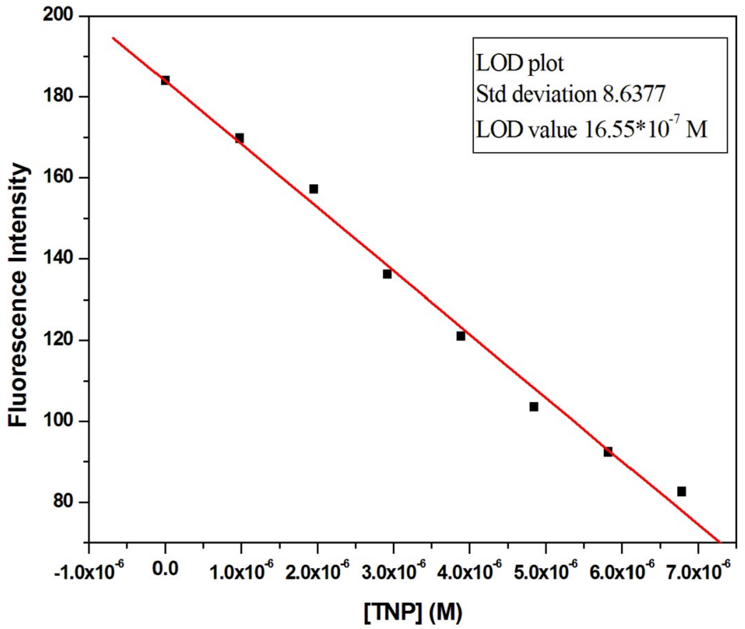

Fig. S9 Limit of detection (LOD) plot for TNP using 3σ method.

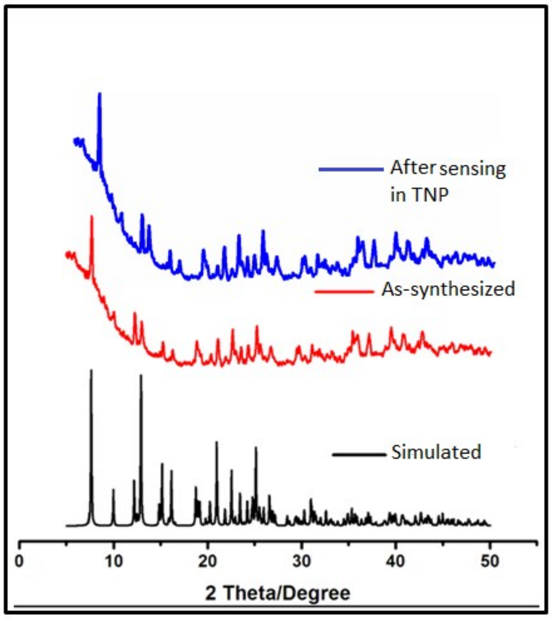

Fig. S10 PXRD patterns of simulated **1** (black), as-synthesized **1** (red) and after sensing of **1** in TNP (blue).

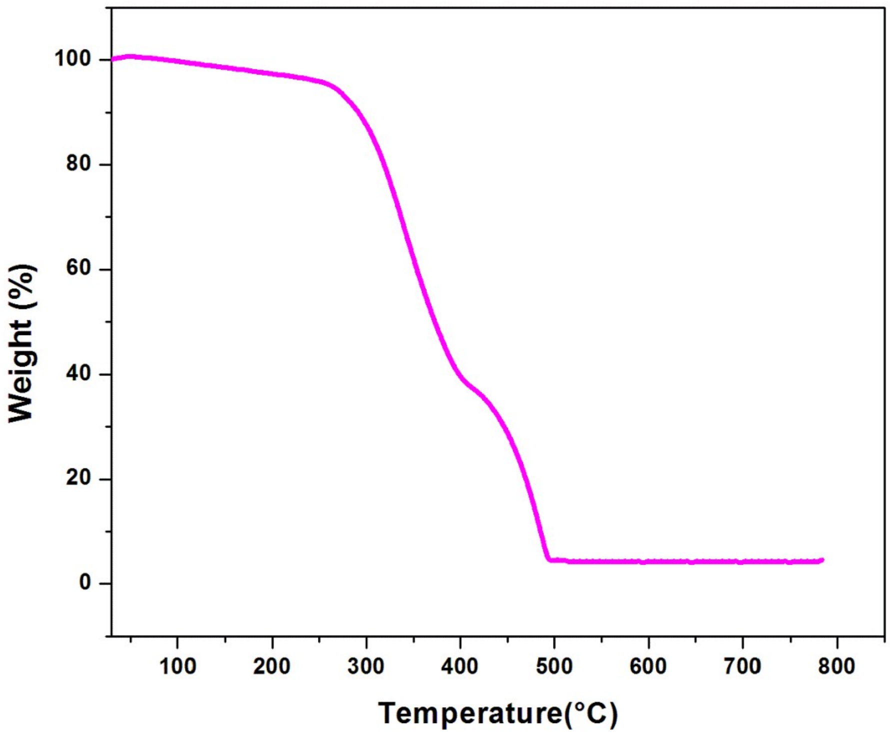

Fig. S11 TGA plot of **1** (pink).
